# Active and Healthy Ageing and Independent Living

**DOI:** 10.1155/2015/542183

**Published:** 2015-08-06

**Authors:** Maddalena Illario, Miriam Vollenbroek-Hutten, David William Molloy, Enrica Menditto, Guido Iaccarino, Patrik Eklund

**Affiliations:** ^1^Department of Translational Medical Sciences, Federico II University and R&D, Federico II University Hospital, Italy; ^2^Roessingh Research and Development, Telemedicine Group, Enschede, Netherlands; ^3^University of Twente, Faculty of Electrical Engineering, Mathematics and Computer Science, Telemedicine Group, Enschede, Netherlands; ^4^Centre for Gerontology and Rehabilitation, St. Finbarr's Hospital, University College Cork, Ireland; ^5^CIRFF, Center of Pharmacoeconomics, Federico II University, Naples, Italy; ^6^Department of Medicine and Surgery, University of Salerno, Salerno, Italy; ^7^Department of Computing Science, Umeå University, Sweden

The recent economic crisis and the current epidemiological trends of Western populations highlighted how the current healthcare systems are inadequate to respond to the evolution of population needs. Among the most important societal challenges is aging, which often is associated with frailty, chronic diseases, and increased multimorbidity. The future costs of an aging society will not be sustainable in terms of healthcare and social services, unless they are reorganized with a focus on prevention and health promotion, in an integrated system. Innovation will be the driver able to turn a burden into an opportunity.

This special issue provides examples of innovative, cross-sectorial strategies that contribute directly or indirectly to improving the quality of life for older adults and their closer ones, making our health and social care systems more effecient and sustainable.

The relevance of connecting leisure time and lifestyle changes is highlighted by two contributions to the issue. Leisure engagement tends to decline with old age, and both articles point out the need for motivational drives to stimulate it. In particular, their evidences suggest that encouraging sedentary and inactive older adults, particularly those over age 65, to maintain or increase their overall daily activity, perhaps simply by reducing time spent in sedentary behaviors, has an even greater impact on reducing the demand for health services.

In the article “Is Self-Reported Physical Activity Participation Associated with Lower Health Services Utilization among Older Adults? Cross-Sectional Evidence from the Canadian Community Health Survey,” the authors examined the relationships between leisure-time physical activity (LTPA) and health services utilization (HSU), in a nationally representative sample of community-dwelling older adults. Active 50–65-year-old individuals were 27% less likely to report any GP consultations and had 8% fewer GP consultations, annually, than their inactive peers. Active persons aged 65–79 years, were 18% less likely than inactive respondents to have been hospitalized overnight, in the previous year. Higher levels of LTPA were significantly associated with lower levels of HSU, across all age groups. In conclusion nonleisure PA appeared to be a stronger predictor of all types of HSU, particularly in the two oldest age groups. Focused strategies to reduce the time spent in sedentary activities may have a positive impact on reducing the demand for health services.

Furthermore, increasing health literacy contributes to a more proactive approach on patient's own health conditions and influences the engagement in leisure activities. Supports and barriers in the social, physical, or societal environment could, for example, be a part of interventions but are also shown to influence physical activities and are suggested to be used as predicting factors.

The aim of the review article “Leisure Engagement: Medical Conditions, Mobility Difficulties, and Activity Limitations—A Later Life Perspective” was to investigate the impact of medical conditions, mobility difficulties, and activity limitations on older people's engagement in leisure activities. The most important predictor of leisure engagement abstention, among older people, is the prevalence of activity limitations, whereas mobility difficulties and medical conditions play less important roles. The strong negative association between activity limitations and leisure engagement remains significant, even after we control for individual, sociodemographic characteristics and country. This study provides a window into leisure engagement in later life and factors influencing the magnitude of engagement in leisure activities.

Raising the awareness about adequate lifestyles is important to ensure adherence to activities that help prevent frailty and adverse health outcomes. This topic is studied by two articles, which look from different viewpoints at the factors influencing lifestyles in older adults. Built environment is just one of many factors affecting older adult's opportunities to age actively. Modifications to their surrounding built environments, may be insufficient to the task of overturning their unwillingness to move outdoors on foot because of fear of falls and injuries, traffic, and poorly maintained pedestrian infrastructure, increasing their personal expectations about aging and changing the perceived comfort and convenience of the motor vehicle.

In the study “Active Aging: Exploration into Self-Ratings of “Being Active,” Out-of-Home Physical Activity, and Participation among Older Australian Adults Living in Four Different Settings,” the authors investigated whether self-ratings of “being active” among older people, living in four different settings (major city high and lower density suburbs, a regional city, and a rural area), were associated with out-of-home participation and outdoor physical activity. Self-ratings of “being active” were found to be positively correlated with the number of days older people spent time away from home but unrelated to time traveled by active means (walking and biking). No significant differences in active travel were found between the four study locations, despite differences in their respective built environments. The findings suggest that additional strategies to the creation of “age-friendly” environments are needed if older people are to increase their levels of outdoor physical activity.

Clinical determinants of physical activity include onset of diseases such as osteoporosis and osteoarthrosis, which can determine a decline in time spent performing physical activity and functional fitness and quality of life with consequent increase in sitting time.

The aim of the review article “Functional Fitness and Self-Reported Quality of Life of Older Women Diagnosed with knee osteoarthrosis: A Cross-Sectional Case Control Study” was to evaluate the functional fitness and self-reported quality of life, differences in older people diagnosed with knee osteoarthrosis who participated in health promotion groups. Ninety older women were distributed into two groups: control without osteoarthrosis of the knee (Control, *n* = 40) and a group diagnosed with primary and secondary knee osteoarthrosis with grade II or higher, with definite osteophytes (osteoarthrosis, *n* = 50). No differences were found between ages of groups (Control: 66 ± 7; osteoarthrosis: 67 ± 9 years). Statistical differences were found on all functional fitness parameters investigated. The values of the chair stand test in the osteoarthrosis (13 ± 5; rep) group were different when compared to the Control group (22 ± 5; rep); additionally, there was a deficit in strength in the lower limbs of 40% when the two groups were compared. This data suggests that knee osteoarthrosis, in older women, can promote a decline in time spent performing physical activity and functional fitness. This is accompanied by a decline in quality of life with an increase in sitting time.

Information Communication Technology (ICT) tools provide sustainable and innovative interventions, which can be adapted to support different health needs of older adults, such as prescription adherence, which can be monitored by ICT tools (e.g., through mobile applications), physical training, and cognitive stimulation.

In the article “A Community-Based, Technology-Supported Health Service for Detecting and Preventing Frailty among Older Adults: A Participatory Design Development Process,” the authors discuss the development of a community-based, technology-supported health service for detecting prefrailty and preventing frailty and further functional decline via participatory design with a wide range of stakeholders, such as physicians and local service providers. The result is an innovative service model in which an online platform supports the integration of traditional services with novel, ICT supported tools.

The aim of the article “Self-Assessment of Adherence to Medication: A Case Study in Campania Region Community-Dwelling Population” was to assess a self-reported medication adherence measure in patients selected during a health education and health promotion focused event, held in the Campania region. The study also assessed sociodemographic determinants of adherence. A total of 312 participants were interviewed during the Health Campus event. A total of 187 (59.9%) had low adherence to medications. Pearson's bivariate correlation showed positive association between the MMAS-8 score and gender, educational level, and smoking. A multivariable analysis showed that the level of education and smoking were independent predictors of adherence. Individuals with an average level of education and smoking were found to be more adherent to medication than those with a lower level of education and smoking. In conclusion, the analysis showed very low prescription adherence levels in the interviewed population. The level of education was a relevant predictor associated with that result.

Risk assessment for different factors is very relevant to identify effective preventive measures. In this issue, different examples are provided of relevant risk components for health outcomes.

In the article “Which Part of a Short, Global Risk Assessment, the Risk Instrument for Screening in the Community, Predicts Adverse Healthcare Outcomes?” the authors investigate the contribution that the three components of the RISC (concern, its severity, and the ability of the caregiver network to manage concern) make to the accuracy of the instrument, across its three domains (mental state, activities of daily living (ADL), and medical state), by comparing their accuracy to other assessment instruments in the prospective Community Assessment of Risk and Treatment Strategies study. Risk of hospitalization was difficult to predict. Thus, each component, but particularly the caregiver network, had reasonable accuracy in predicting institutionalization. No subtest or assessment instrument accurately predicted risk of hospitalization.

The article “Sarcopenia Is Associated with High Pulse Pressure in Older Women” shows a relationship between changes in muscle mass and the cardiovascular system, although this relationship needs further studies to be fully elucidated. Sarcopenic older women (*n* = 43) presented higher levels of pulse pressure (PP) (60.3 ± 2.6 mmHg) and lower muscle function (0.5 ± 0.0 m/s) compared with nonsarcopenic subjects (*n* = 87) (53.7 ± 1.5 mmHg; 0.9 ± 0.0 m/s). Linear regression analysis demonstrated a significantly negative association between skeletal muscle index (SMI) and PP levels. Furthermore, sarcopenic older women showed a 3.1-fold increased risk of having higher PP levels compared with nonsarcopenic women (IC = 1.323–7.506). In conclusion, sarcopenic older women showed lower muscle function and higher cardiovascular risk due to increased PP levels compared with nonsarcopenic subjects.

One of the most important risks for adverse health outcomes for older adults is represented by falls.

The review article “Falls Reduction and Exercise Training in an Assisted Living Population” examines the association between the Boston FICSIT (Frailty and Injuries: Cooperative Studies of Intervention Techniques) exercise program (the original exercise program to demonstrate that nursing home residents can increase strength) and falls incidents in an assisted living community. Among 39 participants, 33% (*n* = 13) reported a fall incident. Adults without a fall history reported more time in aerobic (26.30 versus 20.00, *P* value = 0.71) and strength (1.50 versus 0.50, *P* value = 0.01) training sessions compared to those with a fall history. Multivariate models adjusting for covariates illustrated a significant protective association between strength training and fall incidents (OR = 0.25; 95% CI = 0.07, 0.85). In this cross-sectional study, this progressive resistance exercise training program in an assisted living population was associated with a decrease in the number of fall incidents.

The living environment influences the feelings of perceived well-being and is also important to facilitate stimulating activities fitted to older adults. Standardized arrangements for nursing homes bring the risk of depriving older adults of their memories and negatively influence their willingness to stay involved. Instead, residents should have the freedom to live the lives they want to lead and make changes to their room (by adding personal belongings) without hampering the provision of care. In line with such actions, it should be interesting to explore the degree of freedom a nursing home resident has in relation to making modifications to the home environment or private room.

The study “Picture Your Nursing Home: Exploring the Sense of Home of Older Residents through Photography” investigated which factors in the physical and social environment correlate with the sense of home of the residents and which environmental factors are most meaningful. The four themes identified are (1) the physical view; (2) mobility and accessibility; (3) space, place, and personal belongings; and (4) the social environment and activities. A holistic understanding of which features of the built environment are appreciated by the residents can lead to the design and retrofitting of nursing homes that are more in line with personal wishes.

An ageing society is a challenge and an opportunity to evolve our societies in inclusive and caring ecosystems, where our cultural heritage is valued through the memories of our older adults and bridged to the younger population through intergenerational activities that take advantage of the most up-to-date tools and are capable of driving novel value chains.

*Maddalena**  **Illario* *Maddalena**  **Illario* *Miriam**  **Vollenbroek-Hutten* *Miriam**  **Vollenbroek-Hutten* *David**  **William**  **Molloy* *David**  **William**  **Molloy* *Enrica**  **Menditto* *Enrica**  **Menditto* *Guido**  **Iaccarino* *Guido**  **Iaccarino* *Patrik**  **Eklund* *Patrik**  **Eklund*

